# *Pediatric Research* ECI highlight: Matthew S. Pantell

**DOI:** 10.1038/s41390-024-03214-3

**Published:** 2024-04-25

**Authors:** Matthew S. Pantell

**Affiliations:** 1 Division of Pediatric Hospital Medicine, Department of Pediatrics, University of California, San Francisco, CA USA; 2 Center for Health and Community, University of California, San Francisco, CA USA; 3 Social Interventions Research and Evaluation Network, University of California, San Francisco, CA USA


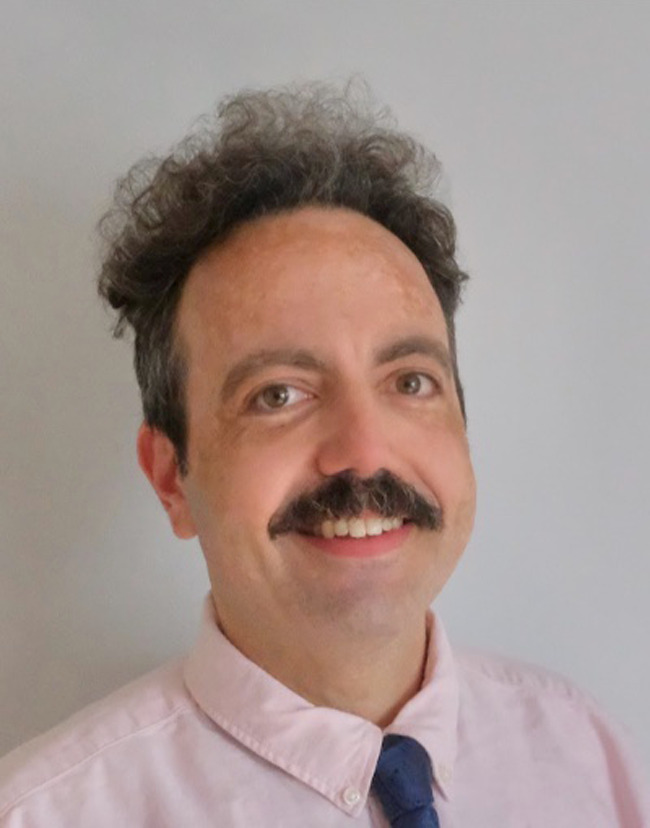


First of all, I would like to say that I am extremely honored to be highlighted as an Early Career Investigator in *Pediatric Research*. I was born and raised in San Francisco, California, where my mother was also born. My father was born in the Bronx and met my mother when moving to San Francisco for a job. My mother worked as a nurse practitioner treating patients living with HIV during the AIDS epidemic at San Francisco General Hospital, the county’s safety net hospital, inspiring my interest in working there many years later.

Before medical school, I attended Oberlin College, a small liberal arts school in Ohio. Majoring in psychology, I became interested in how biological, psychological, and social factors intersect to influence health after taking a health psychology class with my mentor, Dr. Karen Sutton. After college, I worked with Dr. Jenny Kenney at the Health Policy Center of the Urban Institute (UI), a thinktank in Washington, DC.

After the UI, with sustained interest in how societal and policy factors influence health, I attended the University of California (UC) Berkeley – UC San Francisco (UCSF) Joint Medical Program, where I obtained a Master’s in Health and Medical Sciences studying social epidemiology in addition to getting a medical degree. I also met Dr. Nancy Adler, a pioneer in conducting social determinants/drivers of health (SDOH) research. Working with her, I became extremely interested in conducting research on how SDOH are related to health outcomes throughout the lifespan. During medical school, I also pursued a research fellowship at the National Institutes of Health Clinical Research Training Program and began to work with Dr. Laura Gottlieb at UCSF studying how social interventions improve health outcomes.

During my medical school clinical rotations, I realized I belonged in pediatrics. I enjoyed working with children and their families and found it gratifying that addressing health and social issues during childhood could help promote wellness throughout the life course. My current work focuses on: (1) developing best practices for screening for and addressing SDOH in clinical settings; (2) social informatics—exploring how health information technology can be leveraged to integrate social and medical care; and (3) understanding relationships between social factors and health outcomes, including biomarkers. I have a particular interest in exploring relationships between social isolation and loneliness and health.

Nancy passed away this January, and I am forever grateful to have had her in my life. My advice to those beginning a career in pediatric research would be to seek a mentor who is invested in mentoring you, even if their content expertise does not overlap 100%. I am extremely grateful to have found this with Dr. Gottlieb and especially with Dr. Adler, without whom I would not have the research career that I do today.

